# Are we losing the battle against cardiometabolic disease? The case for a paradigm shift in primary prevention

**DOI:** 10.1186/1471-2458-9-64

**Published:** 2009-02-21

**Authors:** Lutz E Kraushaar, Alexander Krämer

**Affiliations:** 1 Department of Public Health Medicine, University of Bielefeld, Bielefeld, Germany

## Abstract

**Background:**

Cardiovascular and diabetic disease are the leading and preventable causes of death worldwide. The currently prognosticated dramatic increase in disease burden over the next two decades, however, bespeaks a low confidence in our prevention ability. This conflicts with the almost enthusiastic reporting of study results, which demonstrate substantial risk reductions secondary to simple lifestyle changes.

**Discussion:**

There is a case to be made for a disregard of the difference between statistical significance and clinical relevance of the reported data. Nevertheless, lifestyle change remains the main weapon in our battle against the epidemic of cardiometabolic disease. But along the way from risk screening to intervention to maintenance the compound inefficiencies of current primary preventive strategies marginalize their impact.

**Summary:**

Unless we dramatically change the ways in which we deploy preventive interventions we will inevitably lose the battle. In this paper we will argue for three provocative strategy changes, namely (a) the disbanding of screening in favor of population-wide enrollment into preventive interventions, (b) the substitution of the current cost utility analysis for a return-on-investment centered appraisal of interventions, and (c) the replacement of standardized programs modeled around acute care by individualized and perpetual interventions.

## Background

Chronic cardiometabolic disease has become a prominent public health concern chiefly for a unique combination of three prospects:

a) pandemic and costly: its constituent diseases lead in the mortality statistics [1-3] and in the top 10 ranks of the most costly diseases [4].

b) predictable: aberrant vital parameters identify at-risk individuals

c) preventable: lifestyle change prevents disease in at-risk individuals

While the first observation is incontestable, the second and third are not. In the following we will challenge some of the notions on which our prevention strategies have been based. We will provide evidence to the fact that our current prevention efforts are inadequate to yield a significant reduction of the epidemic of cardiometabolic diseases. We will argue that our current prevention strategies fail to achieve their objective for three reasons:

1. inefficiencies of screening

2. inefficiencies of intervention

3. inappropriate economic principles

From the discussion of these three aspects we will (a) propose a statistical tool to analyze intervention efficiency and make comparable the results across interventions, and (b) outline the features of an individualized population strategy aimed at a substantial reduction of disease incidence and prevalence.

## Discussion

Methodically sound efforts to investigate the effects of lifestyle modification on the risk of developing chronic disease are relatively recent [5]. Their results are impressive, with investigators reporting such dramatic risk reductions that medical associations now recommend lifestyle change as the primary tool for the prevention of CVD and Diabetes [6,7]. Table 1 lists the ubiquitously quoted randomized case-control studies which investigated the effects of exercise and dietary habit change on the progression from pre-diabetic states to overt diabetes [8-11].

**Table 1:** Diabetes Prevention Studies

***Study***	***Relative risk reduction***
DaQing IGT and Diabetes Study	40%

The Finnish Diabetes Prevention Study (DPS)	58%

The U.S. Diabetes Prevention Program	58%

The Japanese Trial of IGT Males	68%

Exemplary are the DPP and the DPS, each reporting a relative 58% reduction in incident diabetes in subjects with a pre-diabetic impairment of glucose tolerance (IGT) who participated in the trials' lifestyle change intervention arms.

Besides the obvious conclusions these trials are informative for the study and development of preventive strategies for two reasons:

(a) Disease incidence rather than intermediate variables such as risk factors ought to be the outcome measure.

(b) Results should be reported in absolute rather than relative terms, as the former provides for a more realistic grasp of the intervention's impact on disease incidence.

Here is why:

At the end of the three-year DPS study period there was no significant change in fasting plasma glucose (FPG) or post-load glucose (PLG) for either the intervention or the control group [12]. Hidden behind this lack of effect on the intermediate variables of glucose metabolism was a considerable reduction in progression to overt diabetes: Only 8.6% of the subjects of the intervention group had progressed from impaired glucose tolerance (IGT) to overt diabetes vs. 19.8% in the control group. In absolute terms, that is an 11.2% risk reduction, which translates into the more impressive looking relative risk reduction of 58%. That is in congruence with the DPP trial. But, an examination of the latter intervention's effect on CVD demonstrated a significant improvement in risk factors (blood pressure, triglycerides, LDL- and HDL-cholesterol), yet the intervention group's CVD incidents exceeded slightly, though insignificantly, those in the control group [13]. This apparent disconnect between risk factor and disease suggests an insufficient knowledge of the cause-effect relationship between risk parameters and disease outcome. That should show in inefficiencies of disease prediction and risk screening.

### The Inefficiencies of prediction and screening

#### Diabetes

Anderwald et al. investigated the utility of blood glucose values and insulin resistance indices as tools to stratify for diabetes risk [14]. They found no differences in fasted and PLG values between normal controls and apparently healthy offspring of type 2 diabetics who carry a substantially elevated risk for diabetes and its CVD consequences [15].

But the observed difference in peripheral insulin resistance (IR) as well as in insulin mediated suppression of hepatic glucose production [14] does not necessarily make IR a better risk predictor than PLG. IR has been found to predict diabetes risk only in individuals with a family history of the disease but not in those without [16].

#### CVD

In the ARIC study population and in a group of 5,000 British men the metabolic syndrome (MS) was found to approximately double CVD risk [17,18].

However, clinical reality puts statistical significance into perspective: two thirds of all first cases of non-fatal and fatal CVD occurred in those who did not meet the MS diagnostic criteria [17,18].

In the study of Wannamethee et al. the sensitivity and specificity of the MS as a predictor of disease incidence was 0.35 and 0.76 respectively [18]. And 80% of those classified as at-risk were false positives.

That is insufficient for impact. The proportion of CVD that would be prevented in the MS-positive population if the MS were prevented (the attributable proportion in the exposed, APe), and the proportion of CVD that would correspondingly be prevented in the total population (the attributable proportion in the total population, APt) are 33% and 10% respectively. They are achievable only under the unrealistic assumption that no losses occur along the trajectory from risk screening to intervention to post-interventional maintenance. Based on consensus strategies of categorizing as high-risk those with a greater than 20% risk of CVD over the next 10 years [19], the Framingham Risk Score (FRS) does not perform any better than the MS. About two thirds of first incidents of heart disease and stroke happen in individuals whose risk estimation remains below that threshold [18].

A more promising approach may be a neural network based prediction as developed from data of the prospective Cardiovascular Munster Study (PROCAM) [20]. With sensitivity and specificity of 75% and 96% respectively and a false positive rate of only 3% such a screening tool could facilitate a reduction in disease prevalence of about 8%. That's under the assumption that a lifestyle intervention would prevent 11% of cases in the screen-detected at-risk individuals, which is the true prevention rate estimated from prospective modeling of the DPS data [21].

The FRS and PROCAM risk engines are composites of modifiable and un-modifiable parameters, the latter being age, gender and, in the case of PROCAM, family history of CVD. As we cannot change un-modifiable parameters, the utility of these risk engines to gauge the potential effect of risk reduction on incidence rates and prevalence of CVD is limited.

The limited utility of screening has been acknowledged by the U.S. Preventive Services Task Force (USPSTF). With respect to diabetes, the USPSTF considers evidence to be insufficient to recommend for or against routine screening of asymptomatic adults [22]. Screen-selecting high-risk individuals for preventive intervention may, to some degree, be a self-defeating exercise. For an individual the stratification into the risk category that does not require intervention may implicitly endorse a potentially harmful lifestyle which has yet to manifest in clinically relevant parameter aberrations. We thereby a) miss the window of opportunity for marginalizing this potential health threat and b) reinforce detrimental health habits. The latter complicates the future process of lifestyle change once the need for change has become evident from "red-shifted" risk factors.

Thus a public health strategy that builds on risk stratification and prognostication to institute healthy behaviors, may ironically prevent the adoption of such behaviors in the sub-population which currently contributes most of the disease cases.

### Inefficiencies of Intervention

If one defines 'success' as an intervention's ability to produce lasting improvements of dietary and physical activity habits, available promotions and interventions have shown less than encouraging performance.

In the UK the ACTIVE for LIFE campaign which aimed at promoting an increase in physical activity, yielded no measurable behavioral change at a cost of £ 3' Mio [23].

Typically 50% of the participants in physical activity programs will have dropped out by month 6 [24,25]. Among the usually highly motivated participants in clinical trials of popular diets, adherence rates hover around the 50% mark for the initial 12 months [26].

Initial weight loss of 5–10% in obese persons have been reported in weight reduction trials, however with almost entire reversal of weight loss within 3–5 years [27]. In the DPS, less than 40% of the lifestyle participants achieved the weight loss goals, and the gradual regain of weight in those randomized to the DPP lifestyle modification [8] suggests a significant post-interventional decay of adherence to lifestyle change. When defining long-term weight-loss success of maintaining a 10% loss of bodyweight at 12 months, the most optimistic estimates of success rates are 20% [28].

We see three reasons for this high degree of attrition:

1. inadequate consumerization

2. mismatch of chronic need vs. acute provision of intervention

3. neglect of the phenotype

### Inadequate Consumerization

To-date the curricula of lifestyle change interventions have been based on clinical utility and health benefit. Whether and how the interventions appeal to participants has never been factored into their design. The result is a substantial disparity between what would attract at-risk individuals into preventive lifestyle change programs and what these programs offer [29]. This situation frustrates preventive public health efforts in two ways:

• Reducing at-risk individuals' willingness to participate in lifestyle change interventions

• Loss of at-risk individuals to appealing but questionable commercial programs

We posit that the success of a lifestyle change program depends on its curriculum being matched to the targeted individuals' preferences, abilities and environmental constraints.

### Mismatch of chronic need vs. acute provision

Clinical interventions for the prevention of lifestyle disease are modeled around acute care, providing an intervention for a limited period of time. The WHO considers these systems as falling "remarkably short" when tasked with preventing and managing chronic diseases [30]. Given the near constant exposure to the seductions of injurious lifestyles, sustained vigilance is a necessity for sustained success of lifestyle disease prevention.

### Neglect of the phenotype

Treatment guidelines are typically developed from epidemiological and clinical studies which have demonstrated treatment effect by way of intention-to-treat analysis (ITA). ITA evaluates control and intervention subjects as part of the study groups to which they had originally been randomized. As randomization provides for similar baseline profiles across groups, outcome differences between groups can be attributed to the study intervention.

ITA however does neither preclude inter-individual differences in treatment response nor does it make them show. Aberrant response phenotypes may indeed cluster into subgroups whose response to treatment varies due to differences in genetic and epigenetic parameters. This effect can be substantial as evidenced by the fact that only 25–60% of patients benefit from most major drugs [31].

Lifestyle interventions are not different:

1. Physical fitness: Increasing maximal oxygen consumption (VO_2_max) through aerobic activity is a key objective of physical activity interventions aimed at improving cardiovascular fitness. The yield ranges from dramatic improvement to no improvement at all [32]. Moreover, those individuals whose VO_2_max does not respond to aerobic training, may benefit from resistance training [33].

2. Blood pressure: One notable result of the Dietary Approaches to Stop Hypertension (DASH) program were the substantial inter-individually different blood pressure responses to the depletion or repletion of sodium [34]. Correspondingly, while reductions in sodium intake have yielded only marginal reductions in the general population, some individuals show dramatic improvements [35].

3. Body weight: In a 12-weeks volume-controlled physical activity intervention aimed at reducing weight in 30 obese subjects [36], the achieved mean weight loss masked substantial differences between individuals, ranging from 15 Kg weight reduction to slight weight gain. Variations in (a) compensatory energy intake and (b) metabolic response produced large differences in losses of fat and lean mass, all of which remain hidden when data are reported as group means only.

4. The fallacious "obese = risk" equation: Among the (long-term) obese there exists a sub-group with normal metabolic profiles, the metabolically healthy obese (MHO). Correspondingly, the metabolically obese normal weight person (MONW) presents with the biochemical hallmarks of obesity but a normal body weight [37,38]. The MHOs account for up-to one third of the obese population and close to one in 4 normal-weight persons presents with metabolic aberrations [38,39]. Disregard of these phenotypical differences makes weight-based selection strategies miss a substantial proportion of at-risk individuals while wasting resources on those who don't need them.

In the case of the individual patient with an "aberrant" profile, medical/preventive intervention in accordance with ITA informed guidelines will not only fail but also potentially breed frustration and rejection of further preventive intervention.

#### In a nutshell

From screening to intervention to maintenance, the proportion of at-risk individuals which remains or drops out of our reach is simply too large to let us make any dent in disease prevalence.

#### This begs the question

could simply treating everybody with lifestyle change interventions be more beneficial than screening many, treating some and thereby losing most? Disregarding the cost factor the idea is not so far fetched. First, the aspects of lifestyle interventions are consistent with current lifestyle recommendations to the general public. Second, the diagnostics for monitoring the effects of lifestyle change and optimizing the intervention are the same as the screening diagnostics. The idea becomes appealing when the costs for this alternative strategy are equal or less than the costs of our current strategy. And it will spread across populations once the return on investment (ROI) makes population-wide intervention financially attractive to those who pay for and benefit from the health of their population – the health insurers and employers. Obviously cost effectiveness and economic returns are the make-it-or-break-it for this idea.

### Cost effectiveness

The saving grace for the limited reach of our current prevention efforts would be their cost-effectiveness in treated populations. Whether they are cost-effective, we can't say, for two reasons:

• Methodical Inadequacy

• Modeling Uncertainty

#### Methodical Inadequacy

On a societal level the implementation of medical interventions generally follows favorable cost-utility analyses (CUA). CUA compares the relative costs with the relative health gains of different health interventions. The results are typically expressed in dollars spent per quality adjusted life year (QUALY) gained. Since CUA does not attach a monetary value to its outcome measure, it avoids ethical quagmires such as having to decide on the relative values of health of a retired vs. a productively employed person. But this reluctance to express a person's health in dollar terms is a disincentive for developing preventive interventions around favorable return on investment (ROI). The latter would inevitably draw investments from those for whom an individual's health yields a tangible economic benefit – foremost his health insurance agency and his employer. Their willingness to pay for its maintenance is driven by the economic dictum of optimizing profitability. In the German context where (a) health insurance coverage is mandated for every resident and (b) health insurance providers operate under competitive market conditions, it is easy to see how a favorable rate of return would drive the population-wide implementation of preventive interventions. CUA however, not only fails to provide such measure, it can't even give a reliable estimate of what it is supposed to estimate: cost effectiveness. The reason is modeling uncertainty.

#### Modeling Uncertainty

The per-capita cost of implementing the DPP has been estimated at US$ 1,400.- and US$ 700.- (at year 2000 US$ values) for the first and each follow-up year respectively [40]. That translates into cost-estimates ranging from US$ 6,600.- [41] to US$ 62,600.- [21] per QUALY depending on whether Markov or Archimedes modelling strategies are being applied.

Whichever model we prefer, it's an exercise in crystal ball gazing. Hence, little money is being spent for preventive lifestyle change interventions. The OECD, acknowledging that lifestyle has become a stronger determinant for health than the provision of health care itself, allocates only around 3% of its health budgets on preventive interventions [42].

The beneficiaries of people's health need to know the returns on their investments into preventing a deterioration of that health. However, while we don't have the answer, it doesn't mean that we can't get it. What we need to extract it is (a) a new way of post-hoc analysis of available study data (per-compliance analysis) and (b) a modified method of carrying out new studies (population-wide intervention).

### The Need for an Alternative Evaluation Method

Our body of knowledge enables us to formulate lifestyle change interventions which could dramatically reduce disease incidence in any given population [43-49]. Our research objective could therefore broaden from testing the associations between lifestyle change intervention and cardiometabolic health to encompass testing the question whether such interventions "buy" us a reduction in disease incidence at a favorable ROI. That would incorporate an analytical focus on differential analyses of the compliant vs the non-compliant sub-groups. ITA makes it impossible to disentangle the effects of compliance on goal achievement from the inter-group comparison. That is because compliance and non-compliance "happens" in both groups as the DPS trial has demonstrated, where 23% of those who complied with at least 4 of the 5 intervention targets were controls [11]. Controls comply when recruitment strategies attract subjects with a baseline motivation that is high enough to pro-actively change their health behaviors irrespective of their being randomized into the control group.

At given per-capita costs the ROI depends (a) on the intervention's effectiveness at reducing disease incidence in compliant individuals and (b) on the degree of compliance within the population. Obviously, the knowledge about actual ROI lies still far in the future at the conclusion of long-term longitudinal studies to ascertain incidence rate reductions. That does not prevent us from developing and testing interventions which maximize compliance and effect at minimum cost. To compare those new strategies with our current ones we need a slightly different evaluation method. Its purpose will be to demonstrate (a) whether population-wide implementation produces interim surrogate outcome measures (e.g. reduction in risk score or short-term incidence) comparable to those tested in trials such as the DPP at equal or lower costs, and (b) to make such studies of population-wide interventions comparable across context populations.

#### Per-compliance evaluation

While outcome is generally a clearly defined event or an objectively measurable parameter, compliance is a less precise concept. In keeping with this definition and with published elaborations on this subject we define compliance as the degree to which a study subject conforms to a prescribed intervention regimen [50]. To make compliance truly measurable we therefore need to determine cut-off values for the degree of compliance, which we consider the threshold that separates the compliant from the non-compliant subject. Doing so will enable us to apply the evaluation method described in this section to post hoc and comparative analyses of the cost-efficiency of studied and published intervention strategies.

Here is how it works:

To estimate an intervention's effectiveness (E) at producing the target outcome we first need to estimate the outcome that is attributable to the intervention. This is obviously the number of outcome-positive subjects among the compliant participants (O^+^|C^+^) minus the number of outcome-positive subjects among the non-compliant participants (O^+^|C^-^). The effect on the latter cannot be attributed to the intervention. The result is then to be multiplied with the proportion of compliant subjects among the total study population C^+^/ΣS.

E = (O^+^|C^+ ^- O^+^|C^-^) × C^+^/ΣS

E is a dimensionless parameter which facilitates the relative comparison of interventions' effectiveness at achieving similar outcomes. It is inversely proportional to the cost of the intervention per outcome-positive subject whose outcome is attributable to the intervention. Hence, multiplying the inverse of E with the per-capita intervention cost will yield the intervention's per-capita cost for achieving the desired outcome. This facilitates a comparison in economic terms between intervention costs and outcome values across different intervention strategies. It also enables implementation agencies to translate the cost effectiveness findings of any study into their specific context. One has to keep in mind, however, that this value informs about the cost of preventing disease only if disease incidents have been the outcome measure. If surrogate parameters have been used, such as risk factors, the results solely facilitate the comparison of cost efficiencies between interventions.

Tables 2 &3 illustrate the calculations based on a hypothetical study population and on the DPS data [11] respectively.

**Table 2:** Calculation of efficiency

	**outcome +**	**outcome -**	**Total**
**compliant**	56	14	*70*

**non-compliant**	6	24	*30*

**Total**	*62*	*38*	*100*

Tables values are based on the following assumptions:

• sample size *n *= 100 individuals

• compliance is 70%

• 80% of the compliant subjects have the desired outcome

• 20% of the non-compliant subjects have the desired outcome

E = ((56/70) - (6/30)) × (70/100) = 0.42

1/E = 2.38

**Table 3:** Calculation of efficiency based on the DPS data

	**outcome +**	**outcome -**	**Total**
**compliant**	113	4	*117*

**non-compliant**	273	78	*351*

**Total**	*386*	*82*	*468*

the calculations are based on published data of the DPS [11]:

• sample size *n *= 468 individuals

• compliance (having achieved ≥ 3 goals) = 117 individuals

• 113 of the 117 compliant subjects had the desired outcome (no diabetes)

• 273 of the 351 non-compliant subjects had the desired outcome

• compliance in the intervention group: 87 of 235 subjects E = ((113/117) - (273/351)) × (87/235) = 0.07

1/E = 14.3*

*At US$ 2,800 for a 3-year intervention, the prevention of one case of diabetes would cost 2,800 × 14.3 = US$ 40,040

### Consequences for Research

We began this discussion with the question whether our enthusiasm for lifestyle change as a means to reduce the cardiometabolic disease epidemic may be unjustified. We have argued that it is not a lack of effectiveness of lifestyle change per se, rather than an inefficient deployment of screening and intervention strategies which keeps us from winning the battle against this epidemic. As take-home points we have highlighted the need for interventions to be:

• consumerized to participants' expectations

• individualized to their phenotypes and

• perpetualized to counteract the modern environment's constant temptations

However, it will be (a) our ability to create lifestyle change interventions with favorable returns-on-investment and (b) their population-wide application which will decide the outcome of this battle. The former is not in conflict with the tenets of ethics when there is no comparative weighing of individuals' health, and the latter may even be viewed as an ethical imperative. Since it is the consensus view that our current body of knowledge justifies our telling people that prudent lifestyle change will reduce their cardiometabolic risk, the argument may be made that we should discontinue randomizing people into control groups when we know from the outset that their peers in the intervention group will enjoy significantly larger health benefits.

The impetus is on us researchers to develop and test such interventions, and it is on the beneficiaries of individuals' health to facilitate testing them.

The proposition of a population-wide intervention, specifically one that is individualized to each participant's health/risk profile, is usually met with an off-the-cuff objection of being too expensive and too labour intensive. This is not necessarily so. As we have argued above, one major reason for drop-out and post-interventional adherence decay is a lack of concordance between the interventions' curricula and a participant's idiosyncratic abilities, preferences and environmental constraints. It is our experience (from ongoing and yet unpublished work) that tailoring a physical-activity intervention to an individual's idiosyncratic profile is a one-time investment of effort and time, which rewards both, the interventionist and the participant, with substantially increased adherence and effect. If this individualization of the intervention is combined with telemetric adherence monitoring and interactive behavior change technology (IBCT), the interventionist can concentrate on those individuals who require some extra effort, while not wasting it on those who do not need it because they are "going it alone". We currently test the deployment of telemetry and IBCT for individualized preventive interventions on a population level. One of our hypotheses being, that, across a subject population, the inter-individually differing needs for interventionist care will average to a sustainable and cost-efficient level. If so, the results of our work may complement the current investigations into population strategies for disease prevention.

Finland has taken that idea seriously and made it one of three strategies to be tested within the framework of the National Type 2 Diabetes Prevention Programme (FIN-D2D) [51]. The other two being the high-risk strategy and the strategy of early diagnosis and treatment. In the U.S. the DEPLOY pilot study found the delivery of the DPP intervention curriculum through YMCA community centers a promising and feasible alternative to clinical settings. In Germany the National Action Forum Diabetes Mellitus (NAFDM) has adopted the population strategy as part of its national action plan [52]. The European DE-PLAN ("Diabetes in Europe – Prevention using Lifestyle, Physical Activity and Nutritional intervention") project currently tests the feasibility of translating research evidence from prevention studies into efficient population strategies [53].

All these efforts will advance our ability to design and implement a population-wide intervention strategy which effectively reduces disease incidence in a cost-efficient way and without infringing on health care provider resources needed for acute care. Within our German context, we feel that the operationalization does not require expenditure over and above those already being incurred for preventing cardiometabolic disease. Under current laws health insurance agencies partially reimburse members' participation fees for self-selected primary prevention programs. Program selection currently follows no discernible efficiency criteria and is largely left to each individual member's preferences. To substitute this practice for enrolling all members of a regional population into a newly developed intervention for individualized and targeted preventive lifestyle change is what a local health insurer currently tests together in a pilot study with our group. We have designed the intervention to maintain per-capita costs below the annual ceiling of disbursements for preventive efforts. We also deploy telemetric and interactive behavior change technologies to allocate provider intervention exactly as, when and at the intensity required to prevent dropout and to optimize health benefits.

We encourage others to do likewise. The simple tools presented in this paper will facilitate a comparison of performance across populations and designs. To paraphrase the Olympic creed: the most important thing is not to win but to take part.

## Summary

Prudent health behaviors remain the first line of defence against chronic lifestyle disease. But the current format of reducing disease incidence through preventive lifestyle change interventions is plagued by inefficiencies of screening and intervention. We have argued that this inefficiency is due to inadequacies of:

• screening: insufficient knowledge about the cause-effect relationship between acknowledged parameters of disease risk and disease outcome

• intervention: lack of individualization and consumerization

We have consequently proposed

• To substitute a population strategy (i.e. individualized interventions for all) for the high-risk strategy (i.e. standardized interventions to screen selected at-risk individuals).

• To apply a new method of appraising the cost efficiency of preventive interventions for the purposes of making such interventions comparable and to facilitate return-on-investment estimates.

We have reasoned that the costs and efforts for individualization will be more than offset by the resulting increases in participants' adherence to lifestyle change interventions and the long-term maintenance of acquired prudent health behaviors. Additional economies of scale may be realized through the deployment of telemetric and interactive behavior change technology.

We have also argued that only when cardiometabolic health can be expressed in economic terms of return-on-investment will employers and health insurers be willing and able to allocate more resources towards preventive efforts for health maintenance. Once this state of affairs has been reached, the population strategy will gain the momentum of a positive feedback loop that has the potential to finally put a dent into the epidemic of lifestyle disease.

## Competing interests

The authors declare that they have no competing interests.

## Authors' contributions

LK has made substantial contributions to conception and design of this paper, to literature research and writing the manuscript. AK has made substantial contributions to conception and design and to critically review the manuscript, and has given final approval of the version to be published. Both authors have read and approved the final manuscript.

## Pre-publication history

The pre-publication history for this paper can be accessed here:


